# Sex Specific Association of Physical Activity on Proximal Femur BMD in 9 to 10 Year-Old Children

**DOI:** 10.1371/journal.pone.0050657

**Published:** 2012-11-29

**Authors:** Graça Cardadeiro, Fátima Baptista, Rui Ornelas, Kathleen F. Janz, Luís B. Sardinha

**Affiliations:** 1 Exercise and Health Laboratory, Faculty of Human Movement, Technical University of Lisbon, Lisbon, Portugal; 2 Centre of Social Sciences, Department of Physical Education and Sport, University of Madeira, Funchal, Portugal; 3 Department of Health and Human Physiology, Department of Epidemiology, University of Iowa, Iowa City, Iowa, United States of America; Georgia Health Sciences University, United States of America

## Abstract

The results of physical activity (PA) intervention studies suggest that adaptation to mechanical loading at the femoral neck (FN) is weaker in girls than in boys. Less is known about gender differences associated with non-targeted PA levels at the FN or other clinically relevant regions of the proximal femur. Understanding sex-specific relationships between proximal femur sensitivity and mechanical loading during non-targeted PA is critical to planning appropriate public health interventions. We examined sex-specific associations between non-target PA and bone mineral density (BMD) of three sub-regions of the proximal femur in pre- and early-pubertal boys and girls. BMD at the FN, trochanter (TR) and intertrochanter (IT) regions, and lean mass of the whole body were assessed using dual-energy x-ray absorptiometry in 161 girls (age: 9.7±0.3 yrs) and 164 boys (age: 9.7±0.3 yrs). PA was measured using accelerometry. Multiple linear regression analyses (adjusted for body height, total lean mass and pubertal status) revealed that vigorous PA explained 3–5% of the variability in BMD at all three sub-regions in boys. In girls, vigorous PA explained 4% of the variability in IT BMD and 6% in TR BMD. PA did not contribute to the variance in FN BMD in girls. An additional 10 minutes per day of vigorous PA would be expected to result in a ∼1% higher FN, TR, and IT BMD in boys (p<0.05) and a ∼2% higher IT and TR BMD in girls. In conclusion, vigorous PA can be expected to contribute positively to bone health outcomes for boys and girls. However, the association of vigorous PA to sub-regions of the proximal femur varies by sex, such that girlś associations are heterogeneous and the lowest at the FN, but stronger at the TR and the IT, when compared to boys.

## Introduction

Mechanical loading by impact or muscle forces is a contributing factor to skeletal health throughout the life course; however, mechanical loading is particularly important during the transition period from childhood to adolescence. This may be due to the efficient response of bone to loading during middle childhood (elementary school years), since the magnitude of bone accrual associated with mechanical loading is reported to be greater when compared to early childhood and adulthood [1]–[6]. In addition, the evidence shows that the amount and intensity of PA levels are highest during middle childhood compared to other time points across childhood and adolescence [7]. The amount and intensity of PA during middle childhood is important since PA levels dramatically decrease during adolescence [7]–[9]. Consequently, a timely intervention in children’s activity habits when bone appears to be most responsive to activity’s effect and PA is more easily accepted could be an important public health strategy for optimal bone development.

Studies conducted with pre- and early-pubertal children have shown augmented bone mineral accrual in several skeletal sites in both girls [10]–[17] and boys [15]–[17] after high-impact mechanical loading (running and jumping) interventions. The effect of high-impact loading exercise on bone has been shown to be site specific, with accrual occurring at weight bearing sites such as lumbar spine [11] and proximal femur [18]–[22]. The loads imposed during targeted exercise are likely to represent the “best case scenario” and might not generalize to the spontaneous PA choices of children.

Population-based observational studies provide important information on the relationship of bone accrual to the type and amount of PA children voluntarily choose to do. Twenty-five to forty minutes of vigorous PA per day has been suggested as a minimum daily dose for optimal bone growth [23]–[26] but the relationship between bone mineral accrual and PA during the growing years has not been thoroughly examined. Of the studies that exist, there is a lack of consensus on whether sex moderates the association between mechanical loading and bone accrual at the proximal femur [18], [19], [27], [28]. However, it has been suggested that the proximal femur of boys’ is more sensitive to mechanical loading than girls’ [27]. This idea was not derived from work specifically powered to examine sex differences [23], [27]–[31]. Furthermore, most studies reporting a lower responsiveness of PA in girls (when compared to boys) analysed only the femoral neck and not other sub-regions of the proximal femur (i.e. trochanter and intertrochanter).

A potential explanation for sex-specific bone response at the proximal femur is the lower intensity mechanical loading on the skeleton by muscle or impact forces in girls due to their lower lean body mass and less weight-bearing PA. However, given sex differences in body composition [32], [33], in PA [7], [8], maturation timings [34], morphology and gait kinematic parameters [35], it is plausible that bone response sensitivity to PA differs among the proximal femur sub-regions for both boys and girls. This possibility has not been examined and is clinically relevant because women suffer more fragility fractures in old age [36]–[38] and have a higher incidence of fractures at the femoral neck region compared to men who have higher incidence of trochanteric femoral fractures [39], [40]. Therefore, the purpose of this cross-sectional study was to investigate the sex-specific association between non-targeted PA and bone mineral density (BMD) of sub-regions of the proximal femur in boys and girls.

## Methods

### Sample

This cross sectional cohort study included 325 pre and early pubertal subjects (Tanner stage 1 and 2), aged 9–10 years (164 boys and 161 girls) living in the island of Madeira (Portugal) and drawn from the European Youth Heart Study (EYHS). Selection procedures and methods are described in detail elsewhere [24]. None of the subjects were taking any medication affecting bone and none reported a history of bone fracture in lower limbs. The research protocol was in accordance with the Helsinki Declaration. Parents or legal guardians provided written informed consent and the study was approved by the Ethics Committee attached to the scientific board of the Faculty of Human Movement.

**Table 1:** Characteristics of participants as mean±standard deviation.

	Girls (n = 161)	Boys (n = 164)	p*
Age, y	9.7±0.3	9.7±0.3	0.780
Tanner Stage (1/2), %	40/60	4/96	<0.001
Body Weight, kg	34.2±9.0	34.1±7.8	0.960^a,b^
Body Height, cm	137.2±0.1	137.0±0.1	0.813
Body Fat, kg	10.2±5.7	8.2±5.7	0.002^a^
Body Lean Mass, kg	23.1±3.2	25.1±2.9	<0.001
Calcium Intake, mg/d	1020±424	1048±407	0.553
Light PA, min/d	296±47	278±49	0.001
Moderate PA, min/d	142±47	169±55	<0.001
Vigorous PA, min/d	18±14.3	30±21	<0.001^a,b^
Moderate and Vigorous PA, min/d	159±56	198±70	<0.001
Total PA, min/d	456±77	476±90	0.030
PA Average Intensity, count/min/d	586±189	732±273	<0.001
Proximal Femur BMD, g/cm^2^	0.690±0.07	0.753±0.08	<0.001
Femoral Neck BMD, g/cm^2^	0.656±0.06	0.722±0.07	<0.001^b^
Trochanter BMD, g/cm^2^	0.544±0.06	0.591±0.07	<0.001^a,b^
Intertrochanter BMD, g/cm^2^	0.762±0.08	0.820±0.09	<0.001^a,b^
Femoral Neck BMD/Trochanter BMD	1.21±0.08	1.22±0.08	0.095
Femoral Neck BMD/Intertrochanter BMD	0.86±0.05	0.88±0.05	0.001^a^
Trochanter BMD/Intertrochanter BMD	0.72±0.05	0.72±0.05	0.678

* Student’s t-test comparing boys to girls was performed when both variables have normal distribution with the same variance. In cases of no normality or no homogeneity of variances, Mann-Whitney nonparametric test was used.^ a^Girl’s variable without normal distribution; ^b^Boy’s variable without normal distribution. PA - physical activity BMD – bone mineral density.

#### Physical Activity

PA was assessed with a uniaxial accelerometer (model WAM 6471, Manufacturing Technology Incorporated, Fort Walton Beach, FL), over two weekdays and two weekend days. The subjects were asked to wear the accelerometer all day except during water activities, in a representative week of their normal activity, and the procedure was repeated in all cases in which any abnormal event was reported. MAHUffe software (www.mrc-epid.cam.ac.uk) was used to analyze and process activity data. Outcome variables were time (minute/day) spent in light, moderate and vigorous intensity of PA. The intensity of PA was defined according the counts per minute (cpm) as follows: light intensity from 501 to 1999 cpm; moderate intensity from 2000 to 2999 cpm; and vigorous intensity over 2999 cpm. All of the activity data were averaged over the 4-day period and subjects who failed to provide a minimum of 3 days of ≥600 minutes of accelerometer data were excluded. PA procedures are detailed in previous report [41].

**Table 2:** Standardized regression coefficients (β), level of significance (p) and coefficient of determination (R^2^) for proximal femur sub-region models, adjusted for sex, Tanner stage, body height and body lean mass, with data for boys and girls pooled together.

	Predictor Variables	β	p	R^2^
FN BMD	BLM	0.308	<0.001	0.204
	Sex	0.239	<0.001	0.100
	Body Height	−0.064	0.311	–
	Tanner Stage	0.110	0.038	0.009
	Vigorous PA	0.191	<0.001	0.033
	MVPA	−0.004	0.955	–
	Moderate PA	−0.003	0.955	–
TR BMD	BLM	0.372	<0.001	0.209
	Sex	0.170	0.001	0.024
	Body Height	−0.096	0.179	–
	Tanner Stage	0.077	0.156	–
	Vigorous PA	0.227	0.001	0.073
	MVPA	0.039	0.604	–
	Moderate PA	0.031	0.604	–
IT BMD	BLM	0.426	<0.001	0.242
	Sex	0.129	0.012	0.014
	Body Height	0.030	0.634	–
	Tanner Stage	0.082	0.030	–
	Vigorous PA	0.182	<0.001	0.046
	MVPA	0.019	0.798	–
	Moderate PA	0.015	0.798	–

BMD – bone mineral density; FN BMD - femoral neck BMD; TR BMD - trochanter BMD; IT BMD - intertrochanter BMD; PA - physical activity; MVPA – moderate-through-vigorous PA; BLM – body lean mass.

**Table 3:** Standardized regression coefficients (β), level of significance (p) and coefficient of determination (R^2^) for proximal femur sub-region models, adjusted for Tanner stage, body height, and body lean mass, with data for boys and girls treated separately.

	Predictor Variables	Girls			Predictor Variables	Boys		
		β	p	R^2^		β	p	R^2^
FN BMD	BLM	0.483	<0.001	0.233	BLM	0.277	<0.001	0.071
	Body Height	−0.075	0.462	–	Body Height	−0.047	0.639	–
	Tanner Stage	0.100	0.194	–	Tanner Stage	0.100	0.179	–
	Vigorous PA	0.135	0.060	–	Vigorous PA	0.225	0.003	0.051
	MVPA	0.125	0.072	–	MVPA	−0.092	0.440	–
	Moderate PA	0.109	0.116	–	Moderate PA	−0.073	0.440	–
TR BMD	BLM	0.511	<0.001	0.306	BLM	0.238	0.002	0.052
	Body Height	−0.139	0.138	–	Body Height	−0.085	0.407	–
	Tanner Stage	0.062	0.236	–	Tanner Stage	−0.007	0.925	–
	Vigorous PA	0.241	<0.001	0.056	Vigorous PA	0.214	0.005	0.046
	MVPA	0.076	0.400	–	MVPA	−0.016	0.893	–
	Moderate PA	0.064	0.400	–	Moderate PA	−0.013	0.893	–
IT BMD	BLM	0.514	<0.001	0.305	BLM	0.324	<0.001	10.1
	Body Height	−0.017	0.861	–	Body Height	0.061	0.543	–
	Tanner Stage	0.134	0.060	–	Tanner Stage	−0.058	0.434	–
	Vigorous PA	0.213	0.001	0.044	Vigorous PA	0.159	0.033	0.025
	MVPA	0.102	0.263	–	MVPA	−0.063	0.499	–
	Moderate PA	0.086	0.263	–	Moderate PA	−0.081	0.499	–
FNTR BMD	BLM	−0.178	0.024	0.043	BLM	0.027	0.731	–
	Body Height	0.079	0.483	–	Body Height	0.061	0.429	–
	Tanner Stage	0.034	0.690	–	Tanner Stage	0.168	0.031	0.028
	Vigorous PA	−0.172	0.029	0.029	Vigorous PA	−0.033	0.673	–
	MVPA	−0.038	0.730	–	MVPA	−0.072	0.357	–
	Moderate PA	−0.032	0.730	–	Moderate PA	−0.078	0.314	–
					–	–	–	–
FNIT BMD	BLM	−0.250	0.001	0.068	BLM	−0.009	0.932	–
	Body Height	−0.119	0.288	–	Body Height	−0.208	0.006	0.043
	Tanner Stage	0.093	0.272	–	Tanner Stage	0.260	0.001	0.064
	Vigorous PA	−0.095	0.385	–	Vigorous PA	0.057	0.445	–
	MVPA	0.184	0.016	0.029	MVPA	0.041	0.595	–
	Moderate PA	0.313	0.385	–	Moderate PA	0.029	0.706	–
TRIT BMD	–	–	–	–	BLM	0.029	0.781	–
					Body Height	0.223	0.004	0.005
					Tanner Stage	0.076	0.325	–
					Vigorous PA	0.068	0.381	–
					MVPA	0.082	0.296	–
					Moderate PA	0.078	0.324	–

PA - physical activity; BMD – bone mineral density; FN BMD - femoral neck BMD; TR BMD - trochanter BMD; IT BMD - intertrochanter BMD; FNTR – BMD ratio of femoral neck for trochanter; FNIT – BMD ratio of femoral neck for intertrochanter; TRIT- BMD ratio of trochanter for intertrochanter; BLM – body lean mass.

### Clinic Measures

Standing height (to the nearest millimetre) was measured on a stadiometer (Secca 770, Hamburg, Germany) without shoes. Body weight (kilograms), total fat (kilograms), and lean mass without bone (kilograms) were determined from a total body scan by dual x-ray absorptiometry (DXA) (QDR-1500, high-speed performance mode, software 5.7) (Hologic, Waltham, MA; pencil beam, software 5.73). Sexual maturity was assessed using self-report and Tanner’s 5-stage scale for breast development in girls and pubic hair in boys. Children were stratified as prepubertal (Tanner stage 1) or having started puberty (Tanner stage 2) [42].

**Table 4:** Effects of 10 minutes per day of additional physical activity on femoral neck, trochanter, and intertrochanter BMD, adjusted for Tanner stage, body height, and body lean mass.

	β									
	Girls					Boys				
	FN BMD	TR BMD		IT BMD		FN BMD		TR BMD	IT BMD	
ModeratePA	ns	0.00022		0.00031		ns		ns	ns	
		(p = 0.008)		(p = 0.008)						
ModVigPA	0.00014	0.00022		0.00030		0.00015		0.00016	0.00013	
	(p = 0.072)	(p = 0.002)		(p = 0.002)		(p = 0.048)		(p = 0.025)	(p = 0.186)	
VigorousPA	0.00059	0.00101		0.00127		0.00075		0.00056	0.00066	
	(p = 0.056)	(p<0.001)		(p = 0.001)		(p = 0.003)		(p = 0.016)	(p = 0.033)	
	**Δ BMD (%) associated to Δ 10 min/day of physical activity**									
	**Girls**						**Boys**			
	**FN BMD**		**TR BMD**		**IT BMD**		**FN BMD**	**TR BMD**	**IT BMD**	
Moderate PA	ns		0.4		0.4					
ModVig PA	ns		0.4		0.4		0.2	0.3	ns	
Vigorous PA	ns		1.9		1.7		1.0	1.1	0.8	

PA – physical activity; BMD – bone mineral density; FN - femoral neck; TR - trochanter; IT – intertrochanter; ns – non-significant regression coefficient.

BMD from three proximal femur sub-regions, i.e., femoral neck, trochanter and intertrochanter, were measured with DXA (QDR-1500, high-speed performance mode, software 4.76). Quality assurance tests were performed each morning. Precision errors were estimated from 2 measurements in 14 subjects [43]. The coefficients of variation of femoral neck, trochanter and intertrochanter BMD ranged from 1.2% to 1.5%. From the DXA scans, BMD ratios among sub-regions were calculated as indicators of BMD homogeneity in the proximal femur as follows [40]: FNTR is the ratio between femoral neck BMD and trochanter BMD (FNBMD/TRBMD); FNIT is the ratio between femoral neck BMD and intertrochanter BMD (FNBMD/ITBMD); TRIT is the ratio between trochanter BMD and intertrochanter BMD (TRBMD/ITBMD).

Calcium intake were calculated from a semi-quantitative Food Frequency Questionnaire assessing regular intake of a wide set of typical Portuguese foods using the Food Processor SQL software (ESHA Research, Salem OR).

### Statistical Analysis

Data were analysed using the SPSS statistical software package (Version 18.0 for Windows; SPSS, Chicago, IL, USA). Distribution properties of all variables were examined using the Kolmogorov-Smirnov test and appropriate measures of central tendency and variability were selected. Differences between groups (girls and boys) were analysed by Independent-samples T-tests in case of normality and equally of variance and Mann-Whitney nonparametric test otherwise. The Chi-square test of homogeneity was used to compare Tanner stage distributions across sexes. Stepwise regressions were used to analyse associations between PA variables, (i.e., time spent at moderate intensity, vigorous intensity, and moderate-through-vigorous PA - MVPA) and BMD or BMD ratios of proximal femur sub-regions, adjusted for Tanner stage, body height, and total body lean mass. Data were initially analysed with boys and girls pooled together to test the significance of sex as predictor variable and then separately for each sex. All the assumptions for the linear regression analysis were verified (normality and linearity of the residuals, multicollinearity and homoscedasticity). The hypothetical effect of PA intensity on the BMD of the proximal femur sub-regions was estimated by regression analyses (enter approach, p<0.05) calculating the percentage of BMD change associated with an additional 10 minutes per day of PA at two different intensities (MVPA and vigorous PA) by multiplying the unstandardized regression coefficients by 10 and dividing by the correspondent BMD mean at each sub-region of proximal femur. Significance level was set at p<0.05.

## Results

The characteristics of the children are presented in Table 1. There were no differences in age, body weight and height between boys and girls. However, lean mass was higher and fat mass was lower in boys, who were also more active than girls. Boys spent more time in moderate and vigorous PA than girls, whereas girls spent more time in light activities. The proportion of participants in early puberty (Tanner 2) was higher in boys than in girls (40% of the girls and 4% of the boys were in the Tanner stage 1). The BMD of the proximal femur and of its three sub-regions was higher in boys than in girls, but statistically significant sex differences in BMDs ratios were not found, with the exception of the FNIT, with boys revealing a higher ratio than girls.

Associations between PA and BMD of the proximal femur sub-regions were analysed using multiple regression models, first with boys and girls polled together (Table 2) and after separated (Table 3). Among PA variables (time spent at moderate, MVPA, and vigorous PA), vigorous PA was the one with the highest contribution to the R squared of each model (3–7%, p<0.001) (Table 2). None of the other two PA variables showed additional explanatory power once vigorous PA had entered the model. In the same table, body lean mass explained 20–24% of variance in all BMD models (p<0.001) while Tanner stage was responsible for ∼1% variability of femoral neck BMD (p = 0.038). In all the three regression models ran with boys’ and girls’ data pooled together (Table 2), sex turned out to be a significant predictor variable, giving empirical ground for subsequent separated data treatment.

Three other similar models were run for the proximal femur BMD ratios with boys and girls together but none of them complied with the assumptions for regression analysis, having therefore been rejected. Conversely, the models of proximal femur BMD ratios were added to the initial three ones when data was considered separately for boys and girls to analyse associations between BMD and PA variables adjusted for Tanner stage, lean body mass and body height (Table 3). Among all the PA intensity variables examined, vigorous PA was the best predictor: it explained ∼3–5% of the BMD variance (p<0.05) in boy’s femoral neck, trochanter and intertrochanter. However, none of the variation of BMD ratios in boys was predicted by PA intensity variables. In girls, vigorous PA was also the best PA predictor variable explaining 6% of the trochanter BMD and 4% of the intertrochanter BMD variance; a 3% variation in the FNTR and FNIT was also associated with vigorous and MVPA, respectively. In girls, with exception of femoral neck BMD, PA (vigorous and MVPA) explained 3–6% of all BMD variances. Unlike boys, in girls there was a negative association between PA variables and FNTR and FNIT (p<0.05).

Table 3 also shows that body lean mass was a significant predictor variable in all girĺs and boy’s models for the proximal femur’s regional BMDs, except in the girĺs TRIT BMD model.

Table 4 presents regression models using the three PA intensity variables mostly widely used in the literature, moderate PA, vigorous PA and MVPA. In our analysis, there was a higher absolute effect (estimated by unstandardized regression coefficients) of one minute per day of vigorous PA on the BMD than one minute per day of MVPA or moderate PA (only in girls). The effect of PA was not homogeneous for all proximal femur sub-regions and was dissimilar between boys and girls. For example, in girls the hypothetical BMD increase associated with an additional 10 min/day of PA was comparable (∼2%) for trochanter and intertrochanter regions (with no effect on femoral neck). The effect for boys was lower (∼1%) but the response was similar among all three sub-regions of proximal femur.

## Discussion

PA showed a positive contribution to the BMD variation of the three sub-regions of the proximal femur in boys but in girls PA did not help to explain femoral neck BMD variance. For the same duration of PA, the regression coefficients of more intense PA (vigorous PA) were always higher than those corresponding to a less intense PA (MVPA) in boys and girls. The extrapolation of our results suggest a ∼2% higher BMD in the trochanter and intertrochanter regions in girls at the studied age range if an additional 10 minutes per day of vigorous PA is achieved. In boys, the corresponding gain is a ∼1% higher femoral neck, trochanter and intertrochanteric BMDs.

The higher regression coefficients for PA of highest intensity – vigorous PA –compared to lower levels of intensity – MVPA or moderate PA – when regional BMDs of femoral neck, trochanter and intertrochanter are in stake underline the relevance of the PA intensity to bone mineral accrual during the studied pediatric years. The PA threshold under which the effects on bone mass could be modest has been proposed [24]–[26]. Given that boys are usually more active than girls [7]–[9], this could partially explain BMD differences between sexes at proximal femur sub-regions. However this difference seems not be homogeneous among sub-regions. Our results are consistent with studies that reported a positive response of girls’ proximal femur BMD (or bone mineral content - BMC) to PA but also with studies that revealed a response of femoral neck BMD or BMC to PA only in boys. Particularly, our site specific response of girls’ proximal femur in the trochanteric region is in line with the Iowa Bone Development Study which reported 5% and 14% more BMC at the total body and trochanteric region in the most active pre- and early pubertal boys and girls, when compared to inactive peers [29]. Similar effects regarding skeletal regions were also found by Stear et al., who reported greater BMC accrual at the trochanter (4.8%) than in the whole body (0.8%) or lumbar spine (1.9%) in 144 adolescent girls enrolled in a 45-min exercise-to-music classes programme, three times per week, after 15.5-month [44]. Witzke et al. reached analogous findings at the trochanter BMC in adolescent girls using a plyometric jump training programme with no significant differences for the femoral neck, spine or whole body BMC [45]. McKay et al. who examined the effect of an 8-month school-based jumping programme in pre and early pubescent girls, found that the intervention group showed a significantly greater change in trochanter BMD than the control group [16], [18]. Additionally, increments (4.3%) for femoral neck BMC of 8 to 12 years old boys (compared to controls) were reported after 2 years of a high-impact circuit intervention [19]. These observations contradict the idea that girĺs proximal femur is not responsive to PA, although, notably none of these studies reported a positive effect of PA on girl’s femoral neck.

The positive associations that we found between PA and the BMD of the three proximal femur sub-regions in boys and only at the trochanter and at the intertrochanter region in girls is similar with the results of those studies that suggested no effect of PA in girls’ proximal femur, whose analyses were focused in the femoral neck region [6], [10], [11]. The exception, seems to be the study conducted by Petit et al. [17] that showed significant gains in the BMD at the intertrochanter (1.7%) and at the femoral neck (2.6%) region in early pubertal girls (Tanner stages 2 and 3) when compared to controls after a 10-minute jumping programme, 3 times per week during 7 months.

The analysis of all sub-regions of proximal femur in both sexes was a distinctive aspect of our study that provided a more comprehensive examination of bone’s response to PA. Compared to boys, girls showed inferior BMD in the different sub-regions of the proximal femur, which is not new. However, we observed a lower or a tendency to a lower BMD in the femoral neck relative to other sub-regions (FNIT, girls: 0.86 vs. boys: 0.88, p = 0.001; FNTR, girls: 1.21 vs. boys: 1.22, p = 0.095), i.e. the proximal femur sub-region where we did not find any positive association with MVPA or vigorous PA in girls. Our study showed that the pattern of proximal femur responsiveness to PA was more homogeneous in boys, when compared to girls. Conversely, in girls, there were negative relationships between PA and FNTR and FNIT, suggesting a heterogeneous responsiveness favouring the trochanteric and intertrochanteric sub-regions of the proximal femur. If our response pattern findings were generalizable, it is not surprising that researchers using the neck region to represent the entire proximal femur suggest that boys’ femur is more responsive to mechanical loading than girls’ at this age.

In addition to the well-known limitations of DXA technology in the assessment of bone, our study may have an additional limitation due to the self-report of children’s maturity status. The sample selection was based on chronological age (910 yrs) and not to assure a representative maturational profile. At these ages, girls usually demonstrate a more advanced biological maturity than boys which did not happen in our study. However we conducted our analyses with and without adjustments for maturational status obtaining similar results (data not shown).

In conclusion, although a large proportion of bone mineralization is attributable to growth during late childhood, MVPA and especially vigorous PA can have an additional osteogenic effect in the proximal femur. The effect is not homogeneous throughout all bone regions in girls. Our work was not designed to detect why the femoral neck appears non responsive to PA in girls. Further research designed to simultaneously compare site-specific bone responses to PA in boys and girls at a wider age range and level of sexual maturity is needed. In addition, sample sizes should be large enough to allow investigators to test interactions among PA and hip biomechanical factors (as opposed to systemic factors as nutritional, hormonal or sun exposure factors) that can affect differently the BMD of specific regions of proximal femur. Our study shows a region-specific bone response to vigorous PA in pre and early pubertal girls and boys. More active girls have greater BMD in the trochanter and intertrochanter while more active boys have greater BMD in all sub-regions of the proximal femur.
